# DECLINE IN OBJECTIVELY MEASURED STATIC BALANCE WITH AGING

**DOI:** 10.1093/geroni/igad104.2928

**Published:** 2023-12-21

**Authors:** Chitra Banarjee, Renoa Choudhury, Joon-Hyuk Park, Rui Xie, Jeffrey Stout, Ladda Thiamwong

**Affiliations:** University of Central Florida, Orlando, Florida, United States; University of Central Florida, Orlando, Florida, United States; University of Central Florida, Orlando, Florida, United States; University of Central Florida, Orlando, Florida, United States; University of Central Florida, Orlando, Florida, United States; University of Central Florida, Orlando, Florida, United States

## Abstract

Balance performance is a key predictor of falls, contributing to an increased risk of chronic disease and future falls in older adults. In this study, we aimed to characterize the effects of age, gender, and body mass index (BMI) on changes in balance over time. BTrackS, a portable and inexpensive technology system, was utilized to objectively measure the changes in static balance with eyes closed. We assessed 70 community-dwelling older adults (Mage=74.8 years, 77% female, MBMI=26.95 kg/m2) over 2-4 longitudinal observations, with an average of 174 days between baseline and follow-up assessments to characterize the balance performance over time. The balance performance measure was computed as the average center of pressure (COP) path length (cm) across three testing trials. The balance performance measure at baseline significantly positively correlated with age (Pearson’s r=0.417, p< 0.001) and with BMI (r=0.264, p=0.029), but did not differ by gender (p=0.480). Change in balance (difference in static balance over time, cm) was correlated (r=0.393, p< 0.001) with age, such that older adults experienced a greater decline in static balance over time, however, gender (p=0.620) and BMI (p=0.709) did not show a significant correlation. Older adults with increased BMI were found to be more likely to have limitations in balance. These results suggest that clinicians and researchers need to account for the age-related effects on changes in balance performance over time. Rapid deterioration of balance with increased age also has implications for increased monitoring of balance function and further intervention in older adults as they age.

